# Research on Integrated 3D Printing of Microfluidic Chips

**DOI:** 10.3390/mi14071302

**Published:** 2023-06-25

**Authors:** Chuang Wu, Jiju Sun, Binfeng Yin

**Affiliations:** 1 School of mechanical engineering, Yangzhou University, No. 196 West Huang Road, Yangzhou 225127, China; chuangstu@yzu.edu.cn; 2 Nantong Fuleda Vehicle Accessory Component Co., Ltd., Nantong 226005, China; 3 Jiangsu Tongshun Power Technology Co., Ltd., Nantong 226302, China

**Keywords:** microfluidic, 3D printing, bonding method, HIPS, PDMS

## Abstract

Microfluidic chips have the advantages of miniaturization, integration, and portability, and are widely used in the early diagnosis of major diseases, personalized medical treatment, environmental detection, health quarantine, and other fields. The existing microfluidic chip manufacturing process is difficult to operate because of complex three-dimensional channels, complicated manufacturing steps, limited printing materials, the difficulty of operating the bonding process, and the need to purchase expensive new equipment. In this paper, an integrated molding method for microfluidic chips that integrates 3D printing and polymer dissolution technology is proposed. First, the channel mold of poly(vinyl alcohol) (PVA) or high impact polystyrene (HIPS) is dissolved to complete the manufacturing of the microfluidic chip channel. The integrated 3D-forming method of microfluidic chips proposed in this paper can manufacture microchannels inside the microfluidic chip, avoid the bonding process, and eliminate the need for rapid alignment of microchannels, material modification, and other operations, thus improving the stability of the process. Finally, by comparing the microchannels made by PVA and HIPS, it is concluded that the quality of the microchannels made by HIPS is obviously better than that made by PVA. This paper provides a new idea for the fabrication of microfluidic chips and the application of HIPS.

## 1. Introduction

The microfluidic chip can provide a biochemical laboratory on a chip of several square centimeters in size and accurately control fluid through an integrated micro/nanochannel network structure to achieve sample preparation, reaction, separation, monitoring, and other functions. It has the advantages of high sensitivity, high flux, miniaturization, integration, and portability. Microfluidic chips have been widely used in many fields, such as chemistry and medicine [1,2].

The commonly used microfluidic chip processing materials mainly include glass, poly(methyl methacrylate) (PMMA), polydimethylsiloxane (PDMS), silicon chips, etc. [3,4]. Different manufacturing methods are adopted according to different materials, mainly including numerical controlled micromilling technology, photolithography technology, 3D-printing technology, molding technology, etc. [5,6]. CNC-micromilling technology is mainly applied to glass. Carving shapes are designed by two-dimensional drawing software, and then microchannels are processed on glass by programming software to control the path, depth, and spindle speed [7,8]. However, when machining microchannels, it can only be controlled by the feed depth of the tool, and it is impossible to machine three-dimensional microchannels with complex internal structures. Lithography technology uses chemical reactions between chemicals and the surface of the etched material to remove excess materials to form microchannels, which are mainly divided into coating, development, corrosion, cleaning, and other steps [9,10]. The quality of microfluidic chips fabricated by photolithography technology is good, but the steps are too many, the duration is long, and the process is tedious. 3D-printing technology can directly print high-precision and complex substrates with microchannels [11,12], but the available printing materials are limited. Molding technology involves pouring PDMS onto a mold with microchannels, drying and solidifying, demolishing to obtain the substrate with microchannels, then packaging it by bonding, and finally making the inlet and outlet of the microfluidic chip using a PDMS punch. This method is a simple process, environmentally friendly and economical, and suitable for the generation of large quantities of chips [13,14].

At present, the fabrication process of microfluidic chips is mainly divided into the fabrication of microchannels and the packaging of microchannels [15,16,17,18]. When packaging microfluidic chips, the commonly used bonding methods are hot-pressing [19,20,21], plasma [22,23], high-temperature [24,25], and anode [26]. However, hot-pressing bonding requires the use of a polymer hot press for hot pressing, which will inevitably produce slight deformations. At the same time, mature polymer hot presses on the market are small and need to be customized or self-developed [27,28]. Plasma bonding requires rapid and accurate alignment and bonding of microchannels after the surface modification of the base material is completed, which requires high-operation technology [29]. High-temperature bonding requires a muffle furnace to reach temperatures of more than 500 °C, and then annealing to achieve bonding. However, under the effects of high temperature, the microchannels will deform and reduce the bonding accuracy [30,31]. Anodic bonding requires the use of expensive electrostatic-bonding machines to complete bonding [32,33].

To sum up, there are a variety of microfluidic chip fabrication methods at present that can realize the fabrication of microchannels with complex structures and the packaging of microchannels by bonding in the later stage. However, the manufacturing and packaging of microchannels at present have strict requirements on processing equipment and working conditions, and even pose certain risks [34,35]. Therefore, this paper proposes a microfluidic chip processing method that combines 3D-printing technology and polymer-dissolution technology that can form microchannels inside a PDMS substrate. Firstly, the microchannel mold is manufactured by a 3D-printing process; secondly, the microchannel mold is placed in PDMS for packaging; and finally, the 3D-printed microchannel mold is dissolved and removed by solution. The method proposed in this paper combines their respective advantages: it can form microchannels inside the microfluidic chip, omits the bonding process, and does not require rapid alignment of microchannels, material modification, or other operations with high-process requirements, thus improving the stability of the process.

## 2. Materials and Methods

### 2.1. Materials

PDMS (sylgard 184) was obtained from Dow Corning (Midland, MI, USA). High-impact polystyrene (HIPS), poly(vinyl alcohol) (PVA, mw = 1.3 × 105 DA), limonene, and deionized water were purchased from Aladdin biochemical technology, Shanghai, China.

### 2.2. Method

#### 2.2.1. Preparation of Channel Molds

**Figure 1:**
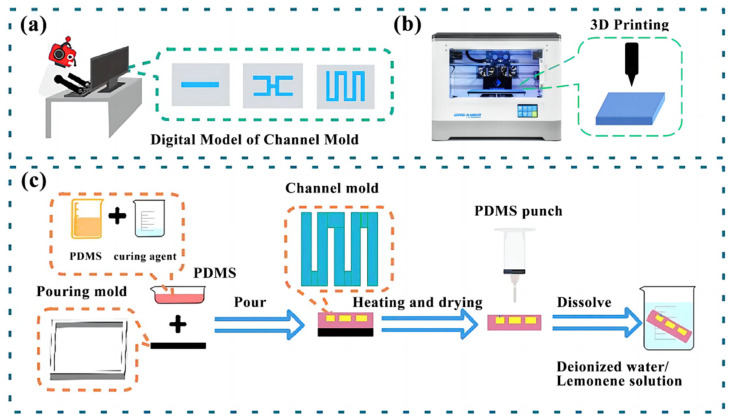
Microfluidic chip manufacturing process: (**a**) 3D-model design of channel mold; (**b**) 3D printing of channel molds; (**c**) Pouring and dissolution of microfluidic chips.

The polymer dissolution method to make microfluidic chips is divided into two parts: the first is to make a dissolvable channel mold through 3D-printing technology, and the second is to remove the channel mold by dissolved liquid. This paper uses PVA or HIPS to manufacture channel molds. The production process is shown in Figure 1. The specific steps are as follows:

Step 1: Mold 3D design. Use the computer-aided design program SolidWorks 2021 to design 3D models of channel molds and casting molds, slice the 3D models, and upload them to a 3D printer (VisiJet^®^ Crystal, 3D Systems) for printing.

Step 2: Mold 3D printing. Since the channel mold needs to be removed by the dissolved liquid in the later stage, it is necessary to set the filling density of the channel mold to 50% and the filling density of the pouring mold to 100%. Otherwise, it will cause the inside of the mold to have a hexagonal pore structure.

Step 3: Prepare the PDMS solution. A curing agent must be added to the PDMS solution before pouring the chip, and the ratio of PDMS to hardener is 10:1.

Step 4: Pour the PDMS substrate of the first layer. Place the PDMS solution in a vacuum dryer to remove the air bubbles generated inside the solution. After removing the bubbles, the PDMS solution is poured on the bottom layer of the mold, and the PDMS is placed in a semi-cured state by drying and heat treatment.

Step 5: Place in the channel mold and encapsulate. After placing the channel mold in the center of the semi-cured PDMS substrate, PDMS solution is poured for encapsulation and cures naturally at room temperature.

Step 6: Dissolve the channel mold. Use a hole punch to punch holes at the top of the channel mold and place it in a dissolving solution to dissolve the channel mold. The dissolving solution, when the channel mold is PVA, is deionized water, and the dissolving solution, when using HIPS, is limonene solution.

#### 2.2.2. Dissolution of Channel Molds

(1) Channel mold of PVA material

PVA will undergo a swelling phenomenon during the process of dissolving in water, and the dissolution of PVA can be accelerated by spontaneously dissolving the PVA. The conditions under which the dissolution can be carried out spontaneously are [36,37]:

ΔG_m_ = ΔH_m_ − TΔS_m_ ≤ 0 (1)

where ΔG_m_ is the mixed free energy; T is the water temperature; ΔH_m_ is the mixed enthalpy; and ΔS_m_ is the mixed entropy.

Four cuboids of PVA with a volume of 1 mm × 1 mm × 10 mm were made to explore the relationship between the dissolution rate and temperature of the PVA channel mold. The cuboids were placed in water at temperatures of 25 °C, 45 °C, 65 °C, and 85 °C to explore the influence of temperature on the dissolution rate of PVA.

(2) Dissolution of HIPS channel mold

Three sets of channel molds with HIPS as the material were made with a 1 mm × 1 mm × 10 mm size to explore the relationship between the dissolution rate of HIPS channel molds and the concentration of limonene. The concentrations of limonene were 0.3 mol/L, 0.5 mol/L, and 1 mol/L, respectively.

#### 2.2.3. Hybrid Experiments with Microfluidic Chips

Microfluidic chips of Y-type channels and T-channels were fabricated using polymer dissolution. PVA was used in the Y-shaped channel, and HIPS was used in the T-shaped channel.

**Figure 2:**
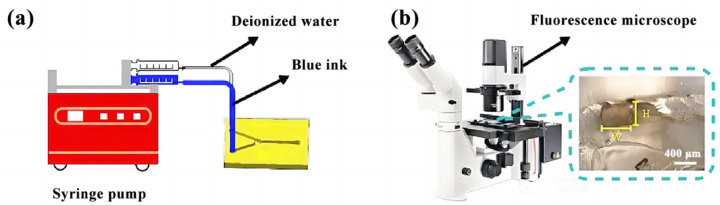
The hybrid experiment of the microfluidic chip: (**a**) schematic diagram of a hybrid experiment; (**b**) observation of channel cross-section by inverted fluorescence microscope.

Two different fluids were used to mix the microfluidic chip to verify the effectiveness of the microfluidic chip. Deionized water and blue ink were injected into the microfluidic chip from different inlets. The solution’s mixing effect was observed by an inverted fluorescence microscope (Eclipse Ti-U, Nikon, Tokyo, Japan) (as shown in Figure 2).

#### 2.2.4. Dimensional Observation of Microchannels

PVA and HIPS were used to make channel molds, fixed position points on the channel molds were selected, and the changes in cross-sectional size, before and after the dissolution, of the fixed position points were observed by fluorescence microscopy to detect the influence of channel mold dissolution on the size of the microfluidic chips.

#### 2.2.5. Comparison of Bonding Methods

In traditional microfluidic chip manufacturing, it is often necessary to use the bonding method for packaging [38,39,40]. For example, PDMS and glass bonding, glass-to-glass bonding, glass-to-PDMS bonding, glass–silicon bonding, PMMA and PMMA bonding, PET and ceramic bonding, etc. For this reason, it is necessary to compare different bonding methods to verify the effectiveness of the proposed microfluidic chip preparation technology.

#### 2.2.6. Statistical Analysis

All data are presented as mean ± one standard deviation (SD) of n samples for each experimental group. Groups were compared using a one-way analysis of variance (ANOVA) to determine the significance. Differences between groups were considered significant when *p* < 0.05.

## 3. Results and Discussion

### 3.1. Channel Mold

**Figure 3:**
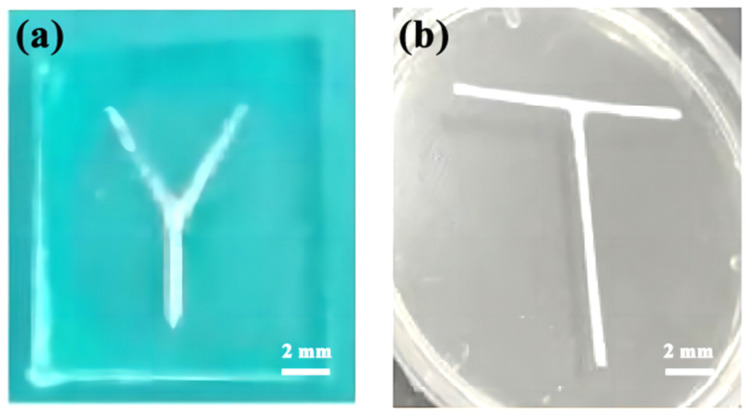
Physical pictures of microfluidic chip: (**a**) Y-type PVA channel mold; (**b**) T-type HIPS channel mold.

The channel mold of PVA was prepared as a Y-type to distinguish the two mold channels of the microfluidic chips. The PDMS microfluidic chip, after pouring, is shown in Figure 3a. The channel mold of HIPS was prepared as a T-type, and the PDMS microfluidic chip was formed after pouring, as shown in Figure 3b.

### 3.2. Dissolution of Mold Channels

(1) Channel mold of PVA

According to Equation (1), water temperature and mixed entropy can directly affect the mixing of free energy. In the full immersion of PVA in deionized water, the water molecules will gradually penetrate the PVA and combine with the hydroxyl group. The PVA expands, the intermolecular force decreases, and the mixed entropy increases. Consequently, the PVA spontaneously dissolves in the aqueous solution.

Since the channel mold of PVA is wrapped inside the PDMS, only the inlet and outlet of the channel can come into contact with water, and sufficient swelling cannot be performed, so the influence of water temperature on the dissolution rate of PVA needs to be considered.

**Figure 4:**
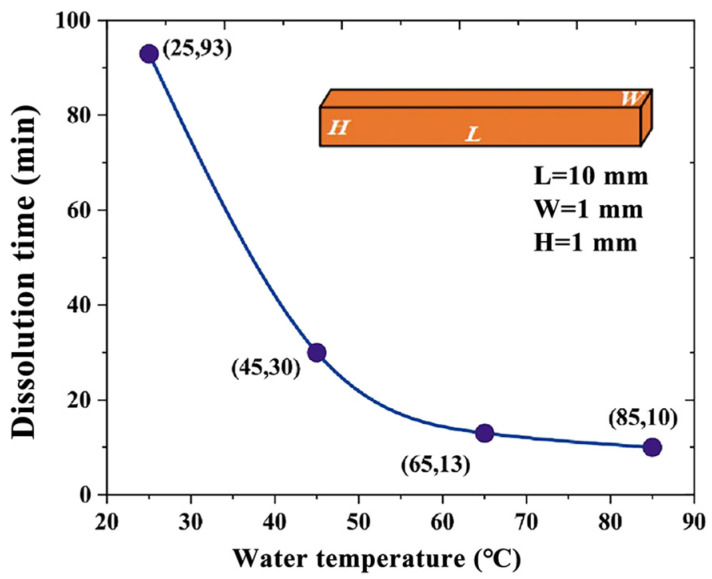
Relation curve between PVA dissolution rate and water temperature.

As shown in Figure 4, the higher the water temperature, the faster the PVA dissolves, and the smaller the acceleration of dissolution. For this reason, 85 °C was selected as the dissolution temperature of the PVA channel mold.

(2) Channel mold of HIPS

**Figure 5:**
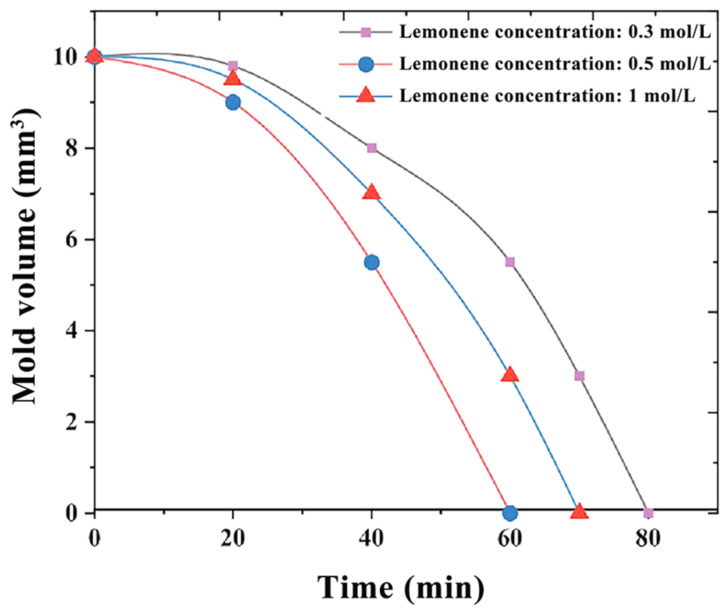
Relation curve between HIPS dissolution rate and limonene solution concentration.

Dissolving HIPS with a limonene solution is an acid-dissolving process. According to Figure 5, when the concentration of limonene solution is 0.5 mol/L, HIPS dissolves the fastest. For this reason, 0.5 mol/L was selected as the concentration of limonene to dissolve the HIPS channel mold.

### 3.3. Hybrid Experiments on Microfluidic Chips

**Figure 6:**
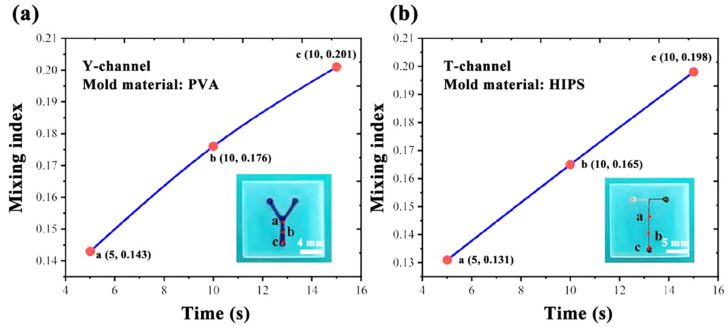
Flowability verification experiment of a microfluidic chip: (**a**) Y-channel of PVA; (**b**) T-channel of HIPS.

Figure 6a shows the mixing efficiency of the channel section of the Y-shaped channel at positions a, b, and c. Figure 6b shows the mixing efficiency of the channel section of the T-channel at positions a, b, and c. As shown in Figure 6, the mixing efficiency of a microfluidic chip made of a Y-type PVA channel mold is 0.143 at point a, increasing to 0.201 at point c. The mixing efficiency of a microfluidic chip made of a T-type HIPS channel mold is 0.131 at point a, increasing to 0.198 at point c.

After the microfluidic chip prepared by the two materials is injected with the two fluids, there are signs of mixing at the final exit, proving that the channel allows flow and verifying the feasibility of the proposed preparation method in this paper.

### 3.4. Size Observation of Microchannels

(1) Channel mold of PVA

**Figure 7:**
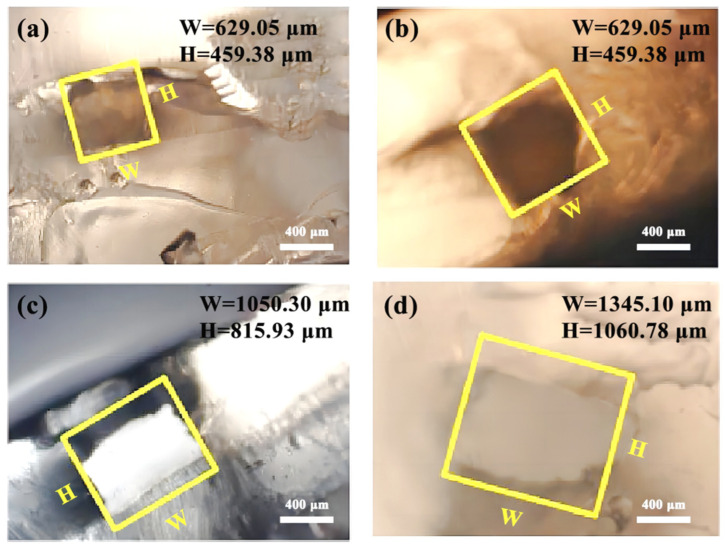
The cross-sectional size after dissolution at four fixed position points on the PVA channel mold: (**a**) cross-sectional dimensions (500, 500) μm; (**b**) cross-sectional dimensions (700, 700) μm; (**c**) cross-sectional dimensions (900, 900) μm; (**d**) cross-sectional dimensions (1200, 1200) μm.

**Figure 8:**
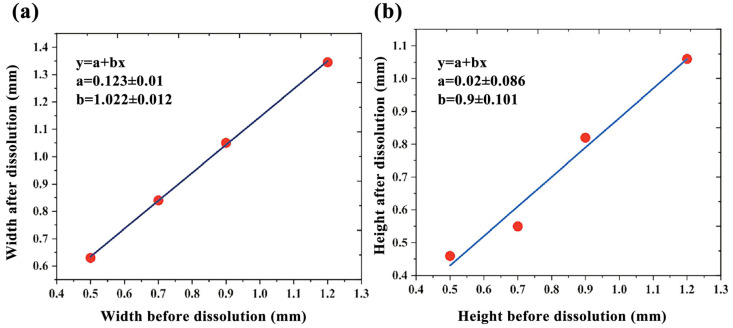
Dimensional error fitting curve before and after the dissolution of a PVA channel mold in a microfluidic chip: (**a**) changes in width before and after dissolution; (**b**) height change before and after the dissolution.

As shown in Figure 7 and Figure 8, the design cross-section widths and heights before dissolution at the four fixed position points on the PVA channel mold were (500, 500) μm, (700, 700) μm, (900, 900) μm, and (1200, 1200) μm. The actual cross-section width and height after dissolution were (629.05, 459.38) μm, (839.96, 546.11) μm, (1050.30, 815.93) μm, and (1345.10, 1060.78) μm.

After dissolution, the channel size of the microfluidic chip changed, the width increased (12–25)%, and the height increased (8–12)%. The main reason is that PVA first expands and then dissolves during the dissolution process [41,42]. During the dissolution process, the small solvent molecule H_2_O diffuses into the PVA macromolecule faster than the rate of PVA dissolution, which will lead to an expansion of PVA volume and an increase in the channel size of the microfluidic chip.

Furthermore, because the dissolution of the PVA channel mold in the microfluidic chip will produce floc, a large external force is required to remove it from the hole, leading to the PDMS chip’s plastic deformation and a reduction of the microchannel’s height.

(2) Channel mold of HIPS

**Figure 9:**
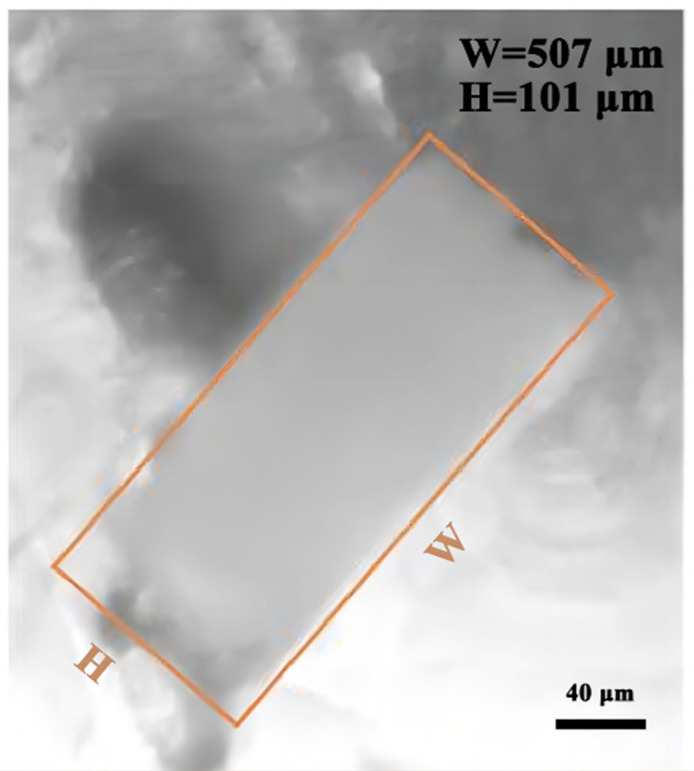
Microchannel cross-section of a HIPS channel mold.

The HIPS channel mold can be directly dissolved due to the acidolysis of the limonene solution [43,44]. The residual HIPS flocculent can be washed off with water without external extrusion, and the size of the dissolved channel changes. As shown in Figure 9, a fixed point was selected: the design section width and height before dissolution were (500, 100) μm, and the actual section width and height after dissolution were (507, 101) μm, respectively.

As shown in Table 1, the dissolved residue of the PVA channel mold needs to be pressed, extruded, and rinsed with clean water. The dissolution residue of the HIPS channel mold can only be washed with water, which has a minor effect on the microfluidic chip size. The dissolution time of both types of materials is about 12 h. The microfluidic section size error before and after the dissolution of the HIPS channel mold is significantly smaller than that of the microfluidic section size error before and after the dissolution of the PVA channel mold. A comparison between the two reveals that the microchannel quality of the microfluidic chip made using HIPS as the channel material and the limonene dissolution method is better.

## 4. Conclusions

In this paper, a Y-shaped channel of a microfluidic chip was made by 3D-printing technology using PVA as the channel material. A T-channel of a microfluidic chip was made by 3D-printing technology using HIPS as the channel material. The relationship between the dissolution rate of PVA and the temperature of water, and the relationship between the dissolution rate of HIPS and the concentration of limonene, were studied. The optimal dissolution temperature of the water was 85 °C, and the optimal concentration of limonene was 0.5 mol/L. Mixing experiments of the Y-channel and T-channel microfluidic chips were conducted. Two different fluids were introduced, and it was found that the mixing efficiency at the outlet position was improved, verifying the microfluidic chip channel fluidity, and ensuring the effectiveness of the microfluidic chip production method integrating 3D-printing technology and polymer-dissolution technology. Finally, by observing the size changes of the two channel materials before and after dissolution using an inverted fluorescence microscope, it was found that the microfluidic chips manufactured using HIPS as the channel molds had the smallest size change and the highest accuracy.

The microfluidic chip prepared in this paper combines the advantages of 3D-printing technology and polymer-dissolution technology, can form microchannels inside the chip, avoids problems such as difficult process manipulation and uneven binding force in the bonding process, improves the stability of the process, has the characteristics of high efficiency and convenience, provides a new idea for the preparation of microfluidic chips and the application of HIPS, and has good application prospects.

## Figures and Tables

**Table 1:** Comparison of PVA channel mold and HIPS channel mold.

Channel Mold Material	Cleaning Method	Dissolution Time	Width Change before and after the Dissolution	Height Change before and after the Dissolution
PVA	Press extrusion	12 h	12–25%	8–12%
HIPS	Clean water flushing	12 h	0.5–1%	1–2%

## Data Availability

Data sharing is not applicable to this article.
